# Bacterial Associates Modify Growth Dynamics of the Dinoflagellate *Gymnodinium catenatum*

**DOI:** 10.3389/fmicb.2017.00670

**Published:** 2017-04-19

**Authors:** Christopher J. S. Bolch, Thaila A. Bejoy, David H. Green

**Affiliations:** ^1^Institute for Marine and Antarctic Studies, University of Tasmania, LauncestonTAS, Australia; ^2^Scottish Association for Marine Science, Scottish Marine InstituteOban, UK

**Keywords:** dinoflagellate, bacteria, interaction, model, *Gymnodinium catenatum*, growth

## Abstract

Marine phytoplankton cells grow in close association with a complex microbial associate community known to affect the growth, behavior, and physiology of the algal host. The relative scale and importance these effects compared to other major factors governing algal cell growth remain unclear. Using algal-bacteria co-culture models based on the toxic dinoflagellate *Gymnodinium catenatum*, we tested the hypothesis that associate bacteria exert an independent effect on host algal cell growth. Batch co-cultures of *G. catenatum* were grown under identical environmental conditions with simplified bacterial communities composed of one-, two-, or three-bacterial associates. Modification of the associate community membership and complexity induced up to four-fold changes in dinoflagellate growth rate, equivalent to the effect of a 5°C change in temperature or an almost six-fold change in light intensity (20–115 moles photons PAR m^-2^ s^-1^). Almost three-fold changes in both stationary phase cell concentration and death rate were also observed. Co-culture with *Roseobacter* sp. DG874 reduced dinoflagellate exponential growth rate and led to a more rapid death rate compared with mixed associate community controls or co-culture with either *Marinobacter* sp. DG879, *Alcanivorax* sp. DG881. In contrast, associate bacteria concentration was positively correlated with dinoflagellate cell concentration during the exponential growth phase, indicating growth was limited by supply of dinoflagellate-derived carbon. Bacterial growth increased rapidly at the onset of declining and stationary phases due to either increasing availability of algal-derived carbon induced by nutrient stress and autolysis, or at mid-log phase in *Roseobacter* co-cultures potentially due to the onset of bacterial-mediated cell lysis. Co-cultures with the three bacterial associates resulted in dinoflagellate and bacterial growth dynamics very similar to more complex mixed bacterial community controls, suggesting that three-way co-cultures are sufficient to model interaction and growth dynamics of more complex communities. This study demonstrates that algal associate bacteria independently modify the growth of the host cell under non-limiting growth conditions and supports the concept that algal–bacterial interactions are an important structuring mechanism in phytoplankton communities.

## Introduction

In natural aquatic systems marine microalgae grow in close association with a complex microbial community (associates) that form an intrinsic component of phytoplankton physiology and ecology (Cole, 1982). Considerable research from a diversity of species indicate that interactions between the associate community and phytoplankton (host) cells are ubiquitous in marine and freshwater systems (Doucette et al., 1998; Amin et al., 2012; Ramanan et al., 2015), play important roles in algal bloom initiation, growth and termination (Azam, 1998; Doucette et al., 1998), and moderate the lifecycle and behavior of algal cells (Adachi et al., 2003; Mayali et al., 2007). Interactions vary from highly specific symbiont/host relationships (e.g., Amin et al., 2015) to commensal/mutualist relationships (e.g., Grossart, 1999; Amin et al., 2009) or parasitic/algicidal behavior (e.g., Fukami et al., 1992; Iwata et al., 2004; Wang et al., 2015), to less-specific interactions such as nutrient competition/modification (Danger et al., 2007). Interactions among associates and the algal host also directly or indirectly alter the behavior, and physiology of both the algal and bacterial partners. For example, phytoplankton stimulate bacteria by supplying much of the organic matter for bacterial growth (e.g., Lau et al., 2007) or produce antibiotics limiting bacterial growth (Cole, 1982). Bacteria produce growth factors such as vitamins and essential nutrients (Croft et al., 2005), increase availability of iron (Amin et al., 2009), or can even modify phycotoxin content and production by diatoms and dinoflagellates (e.g., Osada and Stewart, 1997; Hold et al., 2001; Albinsson et al., 2014).

The composition and structure of associate bacterial communities is broadly similar across different species. For example, among the dinoflagellates, Alphaproteobacteria (Rhodobacteraceae) are the dominant phylotype associated with *Pfiesteria* sp. (Alavi et al., 2001), *Alexandrium tamarense* and *Scrippsiella trochoidea* cultures (Hold et al., 2001). Similarly, members of Gammaproteobacteria belonging to Alteromonadaceae (*Marinobacter* sp. and *Alteromonas* sp.) are associated with a wide variety of dinoflagellates (Alavi et al., 2001; Hold et al., 2001; Seibold et al., 2001; Ferrier et al., 2002; Jasti et al., 2005). Despite the similarities, several studies also indicate that phylogenetically related associates of different phytoplankton species are genetically/functionally different and engage in species-specific interactions (Bolch et al., 2004; Green et al., 2006; Amin et al., 2009).

Associate communities of uni-algal cultures are composed of potentially 1000s of bacterial genotypes; even carefully washed and isolated single cells result in non-axenic cultures with upward of 20–50 bacterial types (Alavi et al., 2001; Hold et al., 2001; Green et al., 2010). This diversity results in potentially 1000s of bacteria–bacteria and bacterial–algal interactions that confound controlled experiments to examine interactions. To address this problem we have developed simplified co-culture experimental models for three dinoflagellate genera (*Scrippsiella, Lingulodinium*, and *Gymnodinium*) that contain a dinoflagellate host and one to three cultured bacterial associates of the dinoflagellate (Bolch et al., 2011). The models provide not only a tractable tool to investigate mechanisms of interaction, but also enable controlled testing of specific hypotheses to gain insight into the function and importance algal–bacterial interactions in complex natural systems.

Despite evidence from culture-based research and evidence of linkages between bacterioplankton and phytoplankton production in nature (Prieto et al., 2015), we have limited knowledge of the relative scale and importance of microbial effects on phytoplankton growth. Models of phytoplankton growth currently include only bottom–up physical factors of light and temperature (Thompson, 1999), availability and uptake of major (C, N, P, and Si) and minor nutrients (Morel and Hudson, 1985), and top-down controls of predation and loss due to sinking (Turner et al., 1998). Here we use the *Gymnodinium catenatum* co-culture model to examine the relative scale and effect of associate bacteria community membership and complexity on dinoflagellate growth dynamics. Our culture experiments indicate that changes in the bacterioplankton community can be as significant for growth of dinoflagellates as changes induced by seasonal changes in light and temperature.

## Materials and Methods

### Bacterial Cultures

Associate bacteria for co-culture experiments were isolated from *G. catenatum* cultures and characterized as detailed in earlier studies (Green et al., 2004, 2010). Three bacterial associates used for co-culture experiments, *Alcanivorax* cf. *borkumensis* DG881, *Marinobacter* sp. DG879 and *Roseobacter* sp. DG874, were selected for experiments based on their ability to support *G. catenatum* growth in uni-bacterial cultures (data not shown). Bacterial cultures were maintained on either Zobell’s marine agar (ZM1) prepared in 75% filtered seawater (26 ppt), or the same medium prepared at 1/10 concentration of nutrients (ZM/10). ZM1 medium contained 5 g L^-1^ of bacterial peptone, 1 g L^-1^ of yeast extract and was solidified with 15 g L^-1^ of Difco-Bacto^TM^ Agar. Both media were supplemented with filter-sterilized trace elements and vitamins (Green et al., 2004). The medium was supplemented with 1% (w/v) sodium acetate as a carbon source when used for *Alcanivorax* DG881 (Green et al., 2004).

### Establishment of Controlled Associate Co-cultures *G. catenatum*

Cultures of *G. catenatum* with specific bacterial associate communities were established following the approach described in detail by Bolch et al. (2011) using resting cysts produced by sexually compatible crosses of *G. catenatum* strains GCHU11 and GCDE08 (hereafter HU11 and DE08 respectively). Briefly, resting cysts of *G. catenatum* were harvested by centrifugation and surface-sterilized by resuspension in 0.5% hydrogen peroxide for 1 h. Batches of 30–40 surface-sterile resting cysts were then aseptically transferred to sterile 36 mm Petri dishes containing 1.9 mL of sterile GSe algal culture medium, a medium based on sterile natural seawater (28 ppt) supplemented with nitrate, phosphate, trace metals, and vitamins (Blackburn et al., 1989). Associate bacterium additions used in the experiments are shown in **Table 1**. Cultures of each associate bacterium were grown overnight in ZM10 medium (Green et al., 2004) and immediately added to dishes of sterile resting cysts in single, pairwise or three-way combinations at a total bacterial concentration of 10^5^ CFU mL^-1^. All bacterial additions were established as independent triplicates. Dishes were sealed with parafilm^TM^ and incubated at 19 ± 2°C at a light intensity of 90 ± 10 μmoles m^-2^ s^-1^ with a 12L:12D photoperiod to allow for resting cyst germination.

**Table 1:** Cultures and bacterial additions used to establish controlled bacterial associate communities.

Culture identifier	Bacterial treatment of resting cysts
M	*Marinobacter* sp. DG879 added to cysts at 10^5^ cells mL^-1^
A	*Alcanivorax* sp. DG881 added to cysts at 10^5^ cells mL^-1^
R	*Roseobacter* sp. DG874 added to cysts at 10^5^ cells mL^-1^
MA	*Marinobacter* sp. DG879 and *Alcanivorax* sp. DG881 in equal proportions; added to cysts at a total concentration of 10^5^ cells mL^-1^
AR	*Alcanivorax* sp. DG881 and *Roseobacter* sp. DG 874 in equal proportions; added to cysts at a total concentration of 10^5^ cells mL^-1^
MR	*Marinobacter* sp. DG879 and *Roseobacter* sp. DG874 in equal proportions; added to cysts at a total concentration of 10^5^ cells mL^-1^
MAR	*Marinobacter* sp. DG879, *Alcanivorax* sp. DG881 and *Roseobacter* sp. DG874 in equal proportions; added to cysts at a total concentration of 10^5^ cells mL^-1^
DEHU	100 μL of 8 μm filtrate from mid-log phase parent cultures GCDE08 and GCHU11; added to cysts undiluted
DE08	Clonal non-axenic parent strain GCDE08 for comparison
HU11	Clonal non-axenic parent strain GCHU11 for comparison

*All cultures were established from 30 to 40 surface-sterilized *Gymnodinium catenatum* resting cysts (GCDE08 × HU11 crosses), germinated and grown for 30 days, transferred to 150 mL Erlenmeyer flasks prior to growth experiments. All culture treatments carried out in duplicate.*

From previous studies (Bolch et al., 2004, 2011; Albinsson et al., 2014), cases of co-culture contamination by non-associate bacteria were detectable during the establishment phase using sterile medium-only controls and in each case resulted from contaminated algal growth medium. To minimize the risk of systematic contamination, all algal culture media was prepared from autoclave-sterilized stock reagents where possible. To control aerial/casual contamination, all co-culture flasks used steristoppers with double-layer foil dust caps and all culture manipulation and transfers were carried out in a class 2 laminar flow cabinet using standard aseptic microbiological techniques. Medium sterility (growth medium only) and cyst sterility controls (cysts with no bacteria added) and mixed parental bacterial community controls were used in all experiments. Sterility controls were assessed for accidental or systematic failure of sterilization or contamination spread plating of multiple undiluted sub-samples onto ZM1 and ZM10 medium (Green et al., 2004). Plates were incubated at 20°C for 7 days, assessed for evidence of bacterial growth by direct visual inspection and 5–60x magnification using a Leica Z9.5 stereomicroscope. If contamination was detected in media-only controls then the experiment was terminated and re-established. If contamination was detected in cyst sterility controls, all cultures derived from the contaminated cyst batch were discarded.

All treatments and control dishes containing resting cysts were examined using a Leica Z9.5 stereomicroscope every 3 days after germination. Cyst quality was monitored by recording final germination (%) and motile dinoflagellate cells by direct examination at 20–63x using a stereomicroscope. After 30 days, two replicates from treatments and positive controls were transferred to sterile 150 mL Erlenmeyer flasks containing 100 mL of sterile GSe medium, stoppered with sterile dust caps to limit aerial or other casual contamination, and grown at 19 ± 2°C under a light intensity of 90 ± 10 μmoles m^-2^ s^-1^ (12L: 12D). Negative control cultures (no added bacteria) resulted in death of *G. catenatum* after germination (see Bolch et al., 2011) and could not be included in further growth studies.

For growth experiments, the established 100 mL cultures were aseptically transferred to 150 ml flasks of sterile GSe medium and grown under the light and temperature conditions described above. Dinoflagellate cell concentration was determined every 4 days from triplicate sub-samples using a Sedgwick-Rafter counting chamber (Guillard, 1973) and by *in vivo* fluorometry (Kiefer, 1973). Bacterial concentration (CFU mL^-1^) was determined every 4 days from triplicate sub-samples and serial dilution spread-plating (Buck and Cleverdon, 1960) onto ZM1 agar. Colony morphology of the associate bacteria used was not sufficiently distinct for reliable differentiation during cell counts therefore total bacterial community counts were undertaken. Bacterial colony morphology on plates was routinely examined for evidence of contamination by non-associate bacteria.

### Statistical Analysis

**FIGURE 1:**
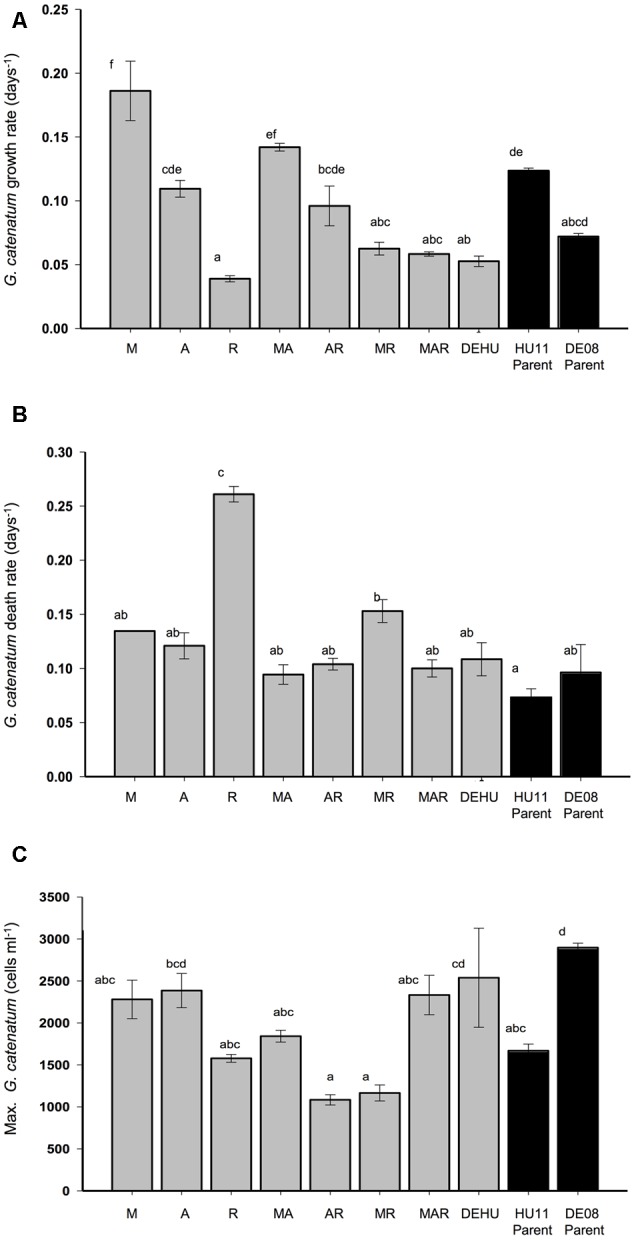
**Exponential growth rate (A)**, death rate **(B)**, and maximum cell density **(C)** (±SE) of *Gymnodinium catenatum* cultures grown with single, pairwise and three-way combinations of bacterial associates compared to mixed culture community (DEHU) and parental non-axenic cultures (GCHU11 and GCDE08). M, *Marinobacter* DG879; A, *Alcanivorax* DG881; R, *Roseobacter* DG874. See **Table 1** for culture codes and additional details. Letters (a–f) indicate significant differences (*p* = < 0.05).

**FIGURE 2:**
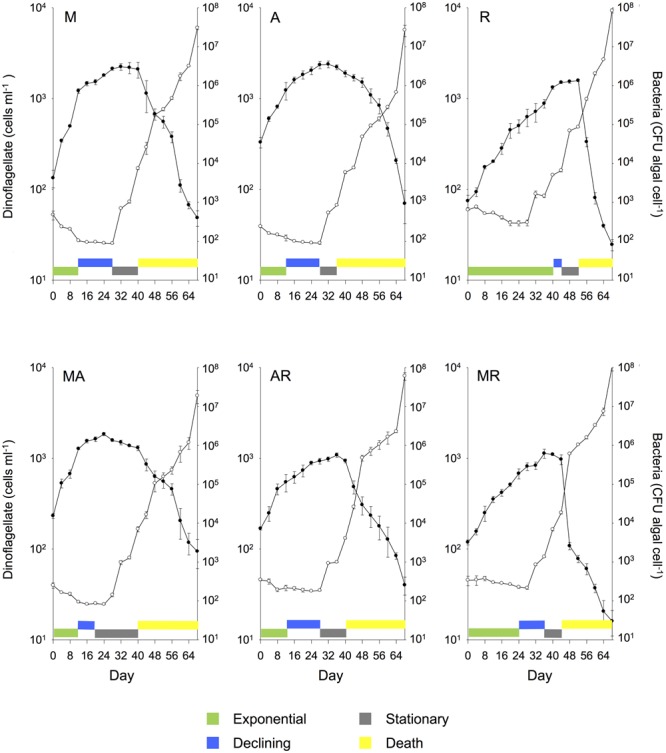
**Mean (± SE) batch culture growth curves of *G. catenatum* (closed circles) and total bacteria (CFU algal cell^-1^, open circles) from duplicate cultures grown with single and pairwise combinations of bacterial associates (M, *Marinobacter* DG879; A, *Alcanivorax* DG881; R, *Roseobacter* DG874; MA, *Marinobacter* DG879 and *Alcanivorax* DG881; AR, *Alcanivorax* DG881 and *Roseobacter* DG874; MR, *Marinobacter* DG879 and *Roseobacter* DG874).** See **Table 1** for culture codes and additional details.

**FIGURE 3:**
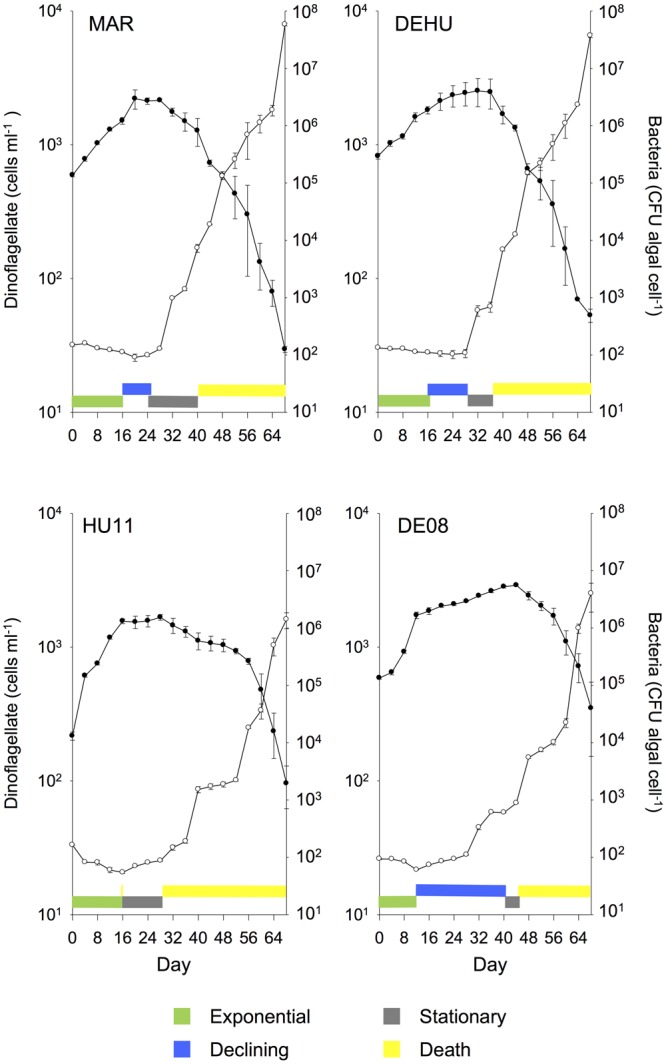
**Mean (± SE) batch culture growth curves of *G. catenatum* (closed circles) and total bacteria (CFU algal cell^-1^, open circles) from duplicate cultures grown with three-way combinations of bacterial associates compared to mixed culture community co-cultures and parent cultures (MAR, *Marinobacter* DG879, *Alcanivorax* DG881 and *Roseobacter* DG874; DEHU, mixed bacterial community from non-axenic GCHU11 and GCDE08 parent cultures; GCDE08, GCHU11, clonal non-axenic parent cultures).** See **Table 1** for culture codes and additional details.

Growth phases were derived from visual inspection of growth curves, and exponential growth/death rates calculated according to Guillard (1973). Differences in dinoflagellate exponential growth and death rates, maximum cell concentration (cells mL^-1^) were compared by one-way ANOVA with significant differences determined by Tukey’s *post hoc* tests, using SPSS ver. 19 (LEAD Technologies, Chicago, IL, USA). Overall similarity of dinoflagellate batch culture dynamics was compared by principal component analysis (PCA) using the software package PRIMER 6. Seven variables were derived from growth curves of each replicate culture for each treatment: exponential growth and death rate (**Figures 1A,B**); maximum cell concentration (**Figure 1C**); and the duration of four batch culture growth phases indicated in **Figures 2, 3**. Variables were normalized prior to analysis (subtraction of variable means, division by variable standard deviation) to account for order of magnitude differences in value ranges (Clarke and Gorley, 2006). The correlation matrix was used for a two-dimensional PCA, and the principal components displayed as an ordination plot.

## Results

### Germination, Sterility, and Negative Controls

No culturable bacteria were detected by dilution spread plating of growth media from sterility controls (medium only) and negative controls (cysts with no bacteria added) indicating that surface-sterilization was effective at removing bacteria and there was low probability of incidental or systematic contamination of media or from airborne sources. Negative controls containing cysts with no bacterial addition showed poor germination rates (15%) and dinoflagellate cells died within the 30 day initial observation period. Long-term culture was not possible and these treatments were not included in the study. All cultures receiving bacterial associate additions exhibited germination rates typical of non-sterilized cysts from earlier studies (54%, Bolch et al., 2002) and similar to the mixed community positive control (DEHU, *p* > 0.066) with the exception of reduced germination in uni-bacterial *Roseobacter* sp. DG874 co-cultures (25%, *p* = 0.013). No significant difference was observed in dinoflagellate cell number per cyst at day 30 post-germination (*f* = 4.422; df = 8, 18; *p* > 0.982).

### Growth Dynamics in Batch Culture

All controlled associate co-cultures were grown successfully to 150 ml flask scale, aseptically transferred, and grown through an extended batch culture cycle over a period of 68 days. Growth curves and rates derived from cell counts were not substantially different to that calculated from *in vivo* fluorescence data (not shown). As fluorescence-based estimates are potentially unreliable outside logarithmic-phase (Falkowski and Kiefer, 1985; Cullen et al., 1988), only cell count data were used for further analysis. Presence or concentration of non-cultured bacteria could not be determined in our experiment, however, routine observation of colony morphology on dilution plates did not detect evidence of contamination by culturable non-associate bacteria in experimental cultures.

Marked differences in dinoflagellate growth rate, death rate, maximum cell concentration (**Figure 1**) and batch culture dynamics were evident between *G. catenatum* cultures grown with different bacterial associate communities (**Figures 2, 3**). No distinct lag-phase was evident, but the exponential growth phase was longer and the stationary phase shorter in cultures containing *Roseobacter* sp., either alone or in combination with other bacteria. Cultures grown with *Marinobacter* sp. or *Alcanivorax* sp. exhibited higher exponential growth rates than mixed associate controls (*f* = 23.99; df = 9,10; *p* = 0.000, 0.033) or cultures containing only *Roseobacter* sp. (*p* < 0.008). Cultures grown with *Roseobacter* sp. showed the slowest exponential growth rate (**Figure 1**) and did not reach stationary phase till day 40–44 (**Figure 2**, R). These co-cultures also exhibited a more rapid decline in death phase than cultures grown with *Marinobacter* sp. (*f* = 19.301; df = 9, 10; *p* = 0.001) or *Alcanivorax* sp. (*f* = 19.301; df = 9, 10; *p* = 0.000) (**Figure 1**).

Co-cultures grown with pair-wise combinations of bacteria exhibited growth curves with a mix of features of the respective uni-bacterial cultures (**Figure 2**, MA, AR, and MR). Co-cultures with *Marinobacter* sp. and *Alcanivorax* sp. showed a short rapid exponential growth period (days 0–12) similar to cultures grown only with *Marinobacter* sp., but a more gradual death phase similar to co-cultures containing only *Alcanivorax* sp. Similar “hybrid” growth curves were evident in the cultures grown with both *Alcanivorax* sp. and *Roseobacter* sp., however, cultures grown with *Marinobacter* sp. and *Roseobacter* sp. showed growth curves similar to co-cultures grown with *Roseobacter* sp. (compare **Figures 2**, MR and R). Mean growth rates of pairwise combinations were intermediate between that of the corresponding uni-bacterial cultures in all cases (**Figure 1**). Mean maximum cell concentrations in two-bacterium co-cultures containing *Roseobacter* sp. achieved a lower maximum cell concentration than mixed community controls (*f* = 6.804; df = 9,10; *p* < 0.01). A sharp decline after day 44 was evident in cultures grown with *Marinobacter* sp. and *Roseobacter* sp., however, the overall rate of decline to day 68 was not different from other two-bacterium combinations.

Cultures grown with communities composed of three bacterial strains (MAR) exhibited batch culture dynamics most similar to those of the mixed associate control (DEHU) containing log-phase bacterial communities from cultures HU11 and DE08 (**Figure 3**). Exponential growth rate, maximum cell concentration and death rates were almost identical (**Figure 1**) and only small differences were noted in the onset and length of batch culture phases (see **Figures 2, 3**). The three-way combination cultures (MAR) exhibited a lower exponential growth rate (*f* = 23.99; df = 9, 10; *p* = 0.041) than either parent crossing strain DE08 and or HU11 (**Figure 1**).

**FIGURE 4:**
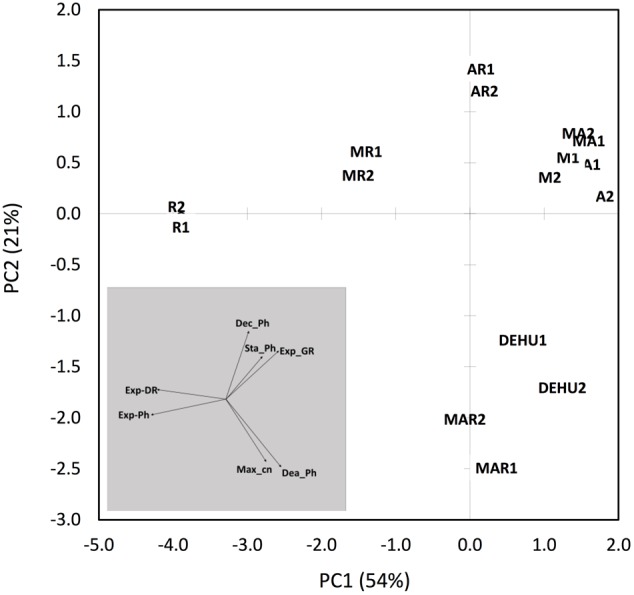
**Two-dimensional principal component analysis (PCA) of dinoflagellate batch growth dynamics of *G. catenatum* cultures grown with different associate bacterial communities.** Variables were normalized prior to analysis and included exponential growth and death rate, maximum cell concentration, and batch growth phase durations indicated by colored bars in **Figures 2, 3**. Replicate co-cultures are indicated by number suffix (1 or 2). See **Table 1** for culture codes and additional detail (M, *Marinobacter* DG879; A, *Alcanivorax* DG881; R, *Roseobacter* DG874; MA, *Marinobacter* DG879 and *Alcanivorax* DG881; AR, *Alcanivorax* DG881 and *Roseobacter* DG874; MR, *Marinobacter* DG879 and *Roseobacter* DG874; MAR, *Marinobacter* DG879, *Alcanivorax* DG881 and *Roseobacter* DG874; DEHU, mixed bacterial community from non-axenic GCHU11 and GCDE08 parent cultures). Vectors of original batch curve variables used in the analysis are shown in the shaded plot. Variables: Dea_Ph, length of death phase in days; Dec_Ph, length of declining phase in days; Exp_DR, death rate (μ) during death phase; Exp_GR, growth rate (μ) during log-phase; Exp_Ph, length of dinoflagellate log-phase in days; Max_cn, maximum dinoflagellate cell concentration (cells ml^-1^); Sta_Ph, length of stationary phase in days.

The two-dimensional PCA of dinoflagellate growth curve parameters separated cultures grown with *Roseobacter* sp. versus *Alcanivorax* sp. and *Marinobacter* sp. along the PC1 axis, primarily due to increased exponential phase duration and increased death rate (**Figure 4**). Pairwise combinations of associates were placed midway between the relevant two uni-bacterial associate cultures. Cultures grown with a three-way associate combination (MAR) and mixed parental associate communities (DEHU) were displaced negatively along the PC2 axis, primarily due to higher maximum cell concentrations and an extended duration of death phase.

### Bacterial Abundance and Growth

Similar patterns of bacterial abundance were observed across all cultures. Bacteria per dinoflagellate cell (CFU dinoflagellate cell^-1^), remained relatively constant during dinoflagellate exponential phase in most cultures, beginning between 100 and 200 bacteria cell^-1^, decreasing to approximately 100 bacteria cell^-1^ by day 24–28 in most cases (see **Figures 2, 3**). Bacteria increased sharply at or near the end of dinoflagellate logarithmic-phase to approximately10^7^–10^8^ bacteria cell^-1^ by day 68 when the experiment was terminated.

**FIGURE 5:**
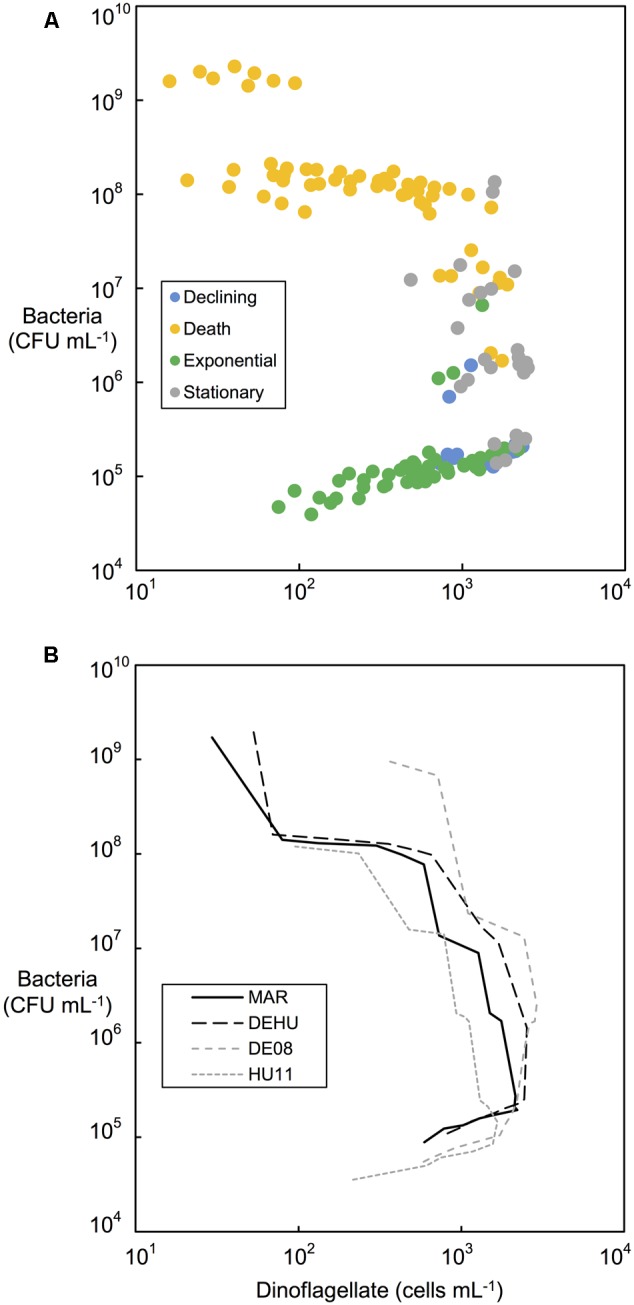
**Bacterial and dinoflagellate cell concentrations (cells mL^-1^) during different phases of dinoflagellate culture growth. (A)** All replicate controlled associate community cultures categorized according to dinoflagellate growth phases indicated by colored bars in **Figures 2, 3**. **(B)** Comparison of mean bacterial and dinoflagellate concentrations from replicate cultures (*n* = 2) of three-way combination (MAR) compared to a mixed community (DEHU) and each parental non-axenic culture (GCDE08 and GCHU11). See **Table 1** for culture codes and additional detail.

**FIGURE 6:**
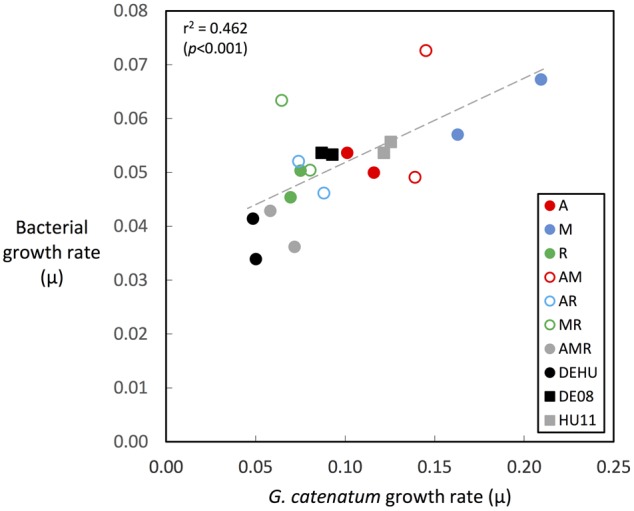
**Correlation of bacterial associate and dinoflagellate growth rates during dinoflagellate exponential phase.** Growth rates were calculated over exponential phase period in each culture as indicated by green-colored bars in **Figures 2, 3** (circles = co-cultures, squares = parent cultures; see **Table 1** for culture codes and additional detail).

Changes in bacterial versus dinoflagellate abundance were strongly associated with dinoflagellate growth phase (**Figures 5, 6**) and followed similar trajectories over the course of the experiment (**Figure 5A**). Bacterial abundance increased with dinoflagellate concentration during dinoflagellate exponential phase, increased rapidly during dinoflagellate stationary phase, and remaining high during culture death phase. Mean abundance patterns differed among treatments, most evident when comparing parental cultures GCDE08 and GCHU11 with the mixed community control (DEHU) and the three-way combination of associates (MAR) which exhibited a similar intermediate microbial-dinoflagellate abundance pattern (**Figure 5B**). Total bacterial community growth rate ranged from 0.03 to 0.075 days^-1^ during dinoflagellate exponential phase and was positively correlated with dinoflagellate growth rate (*r*^2^= 0.46, df = 18, *p* < 0.001; **Figure 6**). From day 28, total bacterial growth rate increased dramatically in all cultures (0.16–0.23 days^-1^), coinciding with onset of stationary phase except in co-cultures with *Roseobacter* sp. and two-way co-cultures containing *Marinobacter* sp. and *Roseobacter* sp. where increased bacterial growth rate coincided with mid- and late-log to declining phase respectively.

## Discussion

**FIGURE 7:**
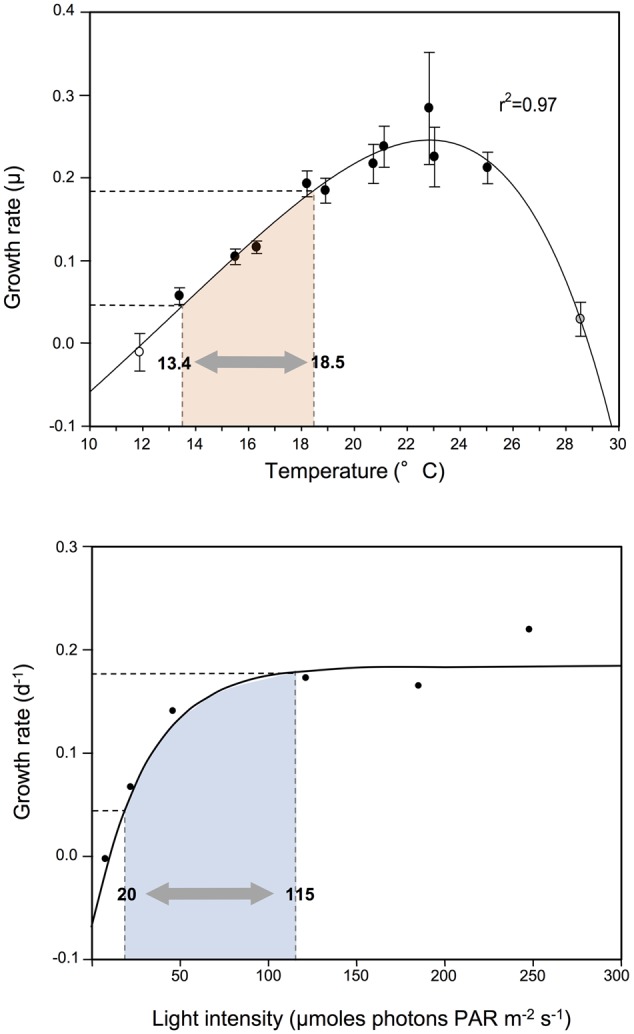
**Changes in exponential growth rate of *G. catenatum* strains due to modified associate bacteria compared to responses to light and temperature.** Range of growth rate change observed due to associate microbial indicated by shaded area within dashed lines; equivalent temperature and light ranges shaded in pink and blue respectively. Temperature and light response of *G. catenatum* at 19°C (equivalent to the present study) were adapted from Armstrong (2010).

Our experiments demonstrate that associate bacterial communities modify dinoflagellate growth independent of other environmental factors considered to control growth of phytoplankton. Co-cultures were grown under identical conditions, in nutrient-replete medium (including vitamins) at saturating light intensity (90–100 μmoles photons PAR m^-2^ s^-1^ at 19°C; Armstrong, 2010), and in the middle of the optimal temperature for *G. catenatum* in both lab culture (12–25°C; Blackburn et al., 1989) and nature (12–20°C; Hallegraeff et al., 2012). The significant observed differences in algal growth dynamics support the concept that the associate bacterial interactions are an important factor in algal population dynamics even under optimal and non-limiting conditions. The scale of change in growth rates was surprisingly large (>four-fold) and equivalent to that typically observed for *G. catenatum* over a five degree temperature range, or an almost six-fold increase/decrease in light intensity (**Figure 7**). Environmental changes of this magnitude are of similar scale to those experienced over an annual cycle in mid-latitude coastal waters of southern Tasmania where *G. catenatum* forms seasonal bloom populations (Hallegraeff et al., 2012), indicating that the influence of bacterial associates is potentially as important for *G. catenatum* population dynamics as light and temperature.

Changes in phytoplankton growth due to modification of bacterial communities are described from a range of phytoplankton species. For example, co-culture with a Flavobacterium increases maximum cell density, growth rate and length of stationary phase of axenic cultures of the diatom, *Chaetoceros gracilis*, and haptophytes *Isochrysis galbana* and *Pavlova lutheri* (Suminto and Hirayama, 1997). Harvestable biomass of the green alga *Botryococcus braunii* increases by 50% when grown in co-culture with a cultured alphaproteobacterial associate (Tanabe et al., 2015). Supplementation with four different associates resulted in a doubling of growth rate and an almost three-fold increase in biomass of *Chlorella vulgaris* (Cho et al., 2015). These findings indicate that bacterial associate interactions are likely to be important for most phytoplankton species.

We observed significant changes in batch culture dynamics, included changes in stationary phase length and cell concentration, and substantial changes in death rate, particularly evident in co-culture with *Roseobacter* sp. DG874. In particular *Roseobacter* co-cultures differed in exhibiting rapidly increasing bacterial concentration from mid-log phase rather than at the onset of stationary phase. The underlying cause or mechanism driving the different growth dynamics is difficult to determine from our data. It may be caused by *G. catenatum* autolysis, or nutrient competition (Wheeler and Kirchman, 1986; Jumars et al., 1989), or alternatively, it may result from a Jekyll-and Hyde interaction where the associate bacterium switches from supportive to algicidal (Seyedsayamdost et al., 2011). Members of the *Roseobacter* clade often dominate the microbial communities associated with phytoplankton and are known to switch from growth promotion to algilytic activity (Geng and Belas, 2010). For example, *Phaeobacter gallaceiencis* produces selective, potent algicides roeseobacticide A and B that lyse *Emiliania huxleyi* (Seyedsayamdost et al., 2011), and *Dinoroseobacter shibae* exhibits similar supportive/algicidal behavior in co-culture with *Prorocentrum minimum* (Wang et al., 2014). The *Roseobacter* in our experiments may also switch to algicidal mode during log-phase dinoflagellate growth, resulting in increased cell lysis that counteracts growth from cell division. This would explain the reduction in net growth rate during mid-log phase and ultimately the 1.5-fold reduction in dinoflagellate concentration at stationary phase. In our models, the mid-log reduction in dinoflagellate growth becomes evident at *Roseobacter* concentrations of 1.5 × 10^5^ cells ml^-1^ (day 20–24), much lower than onset of lysis by *D. shibae* in co-culture models of *P. minimum* (Wang et al., 2014). However, massive cell lysis and steep death phase occurs at similar *Roseobacter* sp. concentrations (10^8^ cells ml^-1^) to that observed in the *Prorocentrum*/*D. shibae* model. Alternatively, the reduction in net-growth rate may be entirely due to *G. catenatum* autolysis (Berges and Falkowski, 1998). The dinoflagellate cannot be grown axenically (Bolch et al., 2011) so we cannot easily determine the extent of autolysis in the absence of bacteria, however there is little evidence of reduced dinoflagellate net growth rate in co-cultures without *Roseobacter* sp., suggesting that autolysis is not the main cause.

Similar promotion/lysis patterns were observed in uni- and mixed-bacterial co-cultures with *Marinobacter* and *Alcanivorax*, however dinoflagellate lysis and decline occurred on or after declining growth phase. In these cultures, cell lysis was more probably stimulated by cell autolysis caused by onset of nutrient stress (Veldhuis et al., 2001) with bacterial algilytic activity perhaps contributing during the subsequent death phase. Previous studies indicate that algicidal activity is cell density-dependent and may be mediated by acetylated homoserine lactones (AHL)-dependent quorum-sensing mechanisms (Paul and Pohnert, 2011; Egan et al., 2013) that up-regulate algilytic compound pathways. Our preliminary analyses of *Marinobacter* genome data indicate that most strains produce either short (C4) or medium (C6-8) AHLs but do not possess a conventional quorum-sensing system (Green, unpublished data). However, it is also possible that algilytic activity is be mediated via other quorum-sensing systems (Bassler, 1999).

We practiced rigorous media preparation processes, careful aseptic technique and sub-sampling to minimize risk of subsequent aerial or other contamination. We did not detect random or systematic contamination of the experiment by culturable non-associate bacteria, but we cannot rule out the presence of uncultured bacteria in our co-cultures. However, the consistency of replicates and clear differences between the associate treatments suggest that if present the effect of uncultured bacteria was either not significant or consistent across the experiment. Studies using the same models/methods described here (Bolch et al., 2004, 2011) indicate that rare instances of co-culture contamination are detectable during the germination and establishment phase and the cultures removed from further experimentation. These studies also cultured associate communities at the end-point of the experiments. Sequencing 16S rDNA of randomly selected isolates routinely recovered only the expected added associates (Bolch et al., 2011).

While *G. catenatum* is considered autotrophic, mixotrophy appears common among photosynthetic dinoflagellates (Jeong et al., 2005) and both intracellular bacteria and bacterial uptake has been reported for this species (Seong et al., 2006). However, bacterial ingestion is estimated to contribute less than 2% to total carbon acquisition by dinoflagellates of similar size to *G. catenatum* (Seong et al., 2006) and is unlikely to have contributed significantly to growth in our co-culture models. Additionally, capacity to support growth in uni-bacterial cultures is limited to only a few bacterial associates and is highly strain/species specific (Bolch et al., 2004). Even closely related bacterial associates to those used here (<0.5% seq. divergence at the 16S rDNA) are unable to support growth in uni-bacterial co-culture (Bolch et al., 2004). Bacterivory cannot explain this high level of specificity. Other experiments indicate that after germination, *G. catenatum* growth can be maintained without bacterial associates at similar growth rates by repeated addition 0.2 μm filtrates from non-axenic log-phase *G. catenatum* cultures (Matsumoto and Bolch, unpublished data). Yet removal of associates from late-log phase co-cultures using antibiotics leads to cessation of growth and ultimately death of the dinoflagellate culture (Bolch et al., 2011). Taken together, these observations indicate that the essential growth factor/s are dissolved or colloidal extracellular products produced by associate bacteria.

The consistent patterns of total bacterial and dinoflagellate concentration, the low bacterial growth rates observed, and the correlated growth rates during dinoflagellate exponential phase indicate that bacterial associate growth was limited by algal-derived organic carbon in our models. During exponential growth, phytoplankton tend to release only a few percent of their photosynthetic products directly (Wiebe and Pomeroy, 1999) therefore supply of organic carbon would logically limit bacterial growth in the co-culture models. This is supported by the low bacterial growth rates we observed during dinoflagellate exponential phase (<0.1 day^-1^) which are 1–2 orders of magnitude lower than associate bacterial taxa grown in organically enriched medium (3.6–8.0 day^-1^ for *Marinobacter* spp.; Guo et al., 2007). Even the fastest bacterial community growth rates observed during dinoflagellate stationary/death phase (0.36–0.42 day^-1^) are at least 10-fold less, suggesting that organic carbon limits associate growth throughout the batch growth cycle.

The uncoupling of bacterial and dinoflagellate growth rates during dinoflagellate stationary and death phases is likely due to onset of algal cell autolysis, resulting in increased supply of organic carbon for bacterial growth and perhaps increased bacterial competition for inorganic nutrients (Bratbak and Thingstad, 1985), further hastening the decline of the dinoflagellate. The stepwise increases in bacterial abundance during these phases have been noted in earlier studies (Bolch et al., 2011) and results from the use of a 12:12 day night cycle which induces synchronous cell division in *G. catenatum* ranging from 4 to 13 days div^-1^ across our experiment. When combined with a 4 day sampling frequency, bacterial growth proceeds in most cultures as a series of rapid increases during/after each semi-synchronous dinoflagellate division. We hypothesize that each division results in quantum increases in host dinoflagellate biomass and exuded organic carbon, which in turn provides substrate for short periods of unconstrained bacterial growth until organic carbon limitation is re-established. The stepwise patterns of total bacterial growth were also remarkably consistent in uni-bacterial and two- and three-way combination models, indicating the same organic carbon limitations and dynamics govern patterns of total abundance of more complex associate communities. We did not track abundance of each bacterial type or assess unculturable bacteria, but other experiments indicate that associates dynamics and behavior in two-way co-culture models can be quite complex. Both the relative proportions of each associate and attachment to algal cell surfaces change markedly over the dinoflagellate growth cycle (Albinsson et al., unpublished), and different associates may interact in different ways during different dinoflagellate growth phases.

Our two- and three-way combination models generally displayed dinoflagellate and bacterial dynamics intermediate of the respective uni-bacterial co-cultures (**Figure 4**). Increasing associate community complexity to three bacteria resulted in dinoflagellate growth dynamics very similar to the more complex mixed-associate controls (DEHU), indicating that our three-way model is sufficient to model interaction and growth dynamics of more complex associate communities. Interestingly, onset of rapid bacterial growth in our MAR co-cultures only occurred in stationary phase, suggesting that the presence of both *Marinobacter* and *Alcanivorax* may moderate the proposed lytic effects of *Roseobacter* sp. DG874. Similar antagonistic interactions among culture associates protect the dinoflagellate *Karenia brevis* from lysis by an algicidal Bacteroidetes bacterium, either through release of specific antibiotic activity, or resource competition stopping the bacterium achieving sufficient concentration for lytic activity (Mayali and Doucette, 2002).

Our previous studies show that *Roseobacter* (including DG874) dominate (85%) the associate community of GCDE08, with *Marinobacter* as sub-dominant (13%) and *Alcanivorax* being relatively uncommon (<1%) (Green et al., 2010). The *Roseobacter*-dominated community may thus explain the distinctive two-phase exponential growth pattern of GCDE08 cultures, also evident to varying degree in other *Roseobacter* co-cultures in this work (**Figures 2, 3**; AR and MR). By logical extension, differing associate community composition may also explain a component of within-species variation in growth/performance commonly observed in algal culture studies. Such observations are usually explained as genetic diversity or uncontrolled/random variance, however, our model indicates that *Roseobacter* dominated associate communities can lead to reduced growth rate and culture yield. The averaging effect seen in combined co-cultures suggest associate-related effects may be moderated when there is sufficient associate diversity/redundancy, but standard phytoplankton isolation techniques involve several cell-washing steps that reduce associate community diversity. As an example, the total number of associate taxa in parent *G. catenatum* cultures in our models differ substantially; seven associate taxa in strain GCHU11 versus 17 in GCDE08 (Green et al., 2010). This is sufficiently low for associate effects to be a significant contributor to strain variation and a confounding factor in culture-based algal growth studies.

The importance and contribution of microbial interactions in phytoplankton population decline and nutrient cycling have been recognized for some time but our study shows that associate microbial interactions are of potential equal importance to the physical factors traditionally thought to moderate phytoplankton growth and primary production in the world’s aquatic ecosystems. Studies of coastal plankton communities have recently shown that phytoplankton production is reliant on a metabolically active heterotrophic bacterial community even when sufficient inorganic nutrients are available for growth (Prieto et al., 2015), demonstrating that the bacterioplankton can be essential for phytoplankton production in nature. Culture-based studies with models like those used here describe a range of mechanisms that may mediate these processes. For example, a metabolically active bacterioplankton community may be essential for phytoplankton trace metal uptake via an interaction process known as Iron-Carbon mutualism (Amin et al., 2009). Algal-associates of the genus *Marinobacter* produce the photo-labile iron siderophore, Vibrioferrin (VF), that is released into the algal cell boundary layer. Photolysis of VF leads to release of soluble Fe^3+^ near the algal cell surface that is rapidly taken up by the algal cell, increasing algal iron uptake rate by almost 20-fold (Amin et al., 2009). The capacity for photo-labile siderophore production is widespread in the natural marine bacterial populations, but estimated to be present in perhaps only 1–2% of the total microbial community (Gardes et al., 2013), as one might expect if this capacity is associated predominantly with the low abundance algal-associate community. The challenge is now to understand the specific conditions under which these interactive mechanisms alter/modify growth and primary production, and the level of functional redundancy that exists in both the associate and background free-living bacterial communities.

## Author Contributions

Authors CB and DG were responsible for the concepts and experimental plan, design of experiments, and the provision of research material including isolation of bacterial strains, and supply of algal strains for the study. Experimental data collection, and analysis was carried out by TB and CB. Drafts of the manuscript, figures and tables were completed by TB with input from CB. The final manuscript and figures were revised by CB with input from DG.

## Conflict of Interest Statement

The authors declare that the research was conducted in the absence of any commercial or financial relationships that could be construed as a potential conflict of interest.
